# Spontaneous Second Molar Migration After MIH-Affected First Molars Extraction: A Radiographic-Based Method Evaluation

**DOI:** 10.3390/children12121589

**Published:** 2025-11-23

**Authors:** Santo Andrea Stabilini, Lucia Giannini, Niccolò Cenzato, Francesca Olivi Mocenigo, Claudia Salerno, Cinzia Maspero, Maria Grazia Cagetti

**Affiliations:** 1 Department of Biomedical, Surgical and Dental Sciences, University of Milan, 20122 Milan, Italy; santo.stabilini@unimi.it (S.A.S.); francesca.olivi@unimi.it (F.O.M.); claudia.salerno@students.unibe.ch (C.S.);; 2 Fondazione IRCCS Cà Granda Ospedale Maggiore Policlinico, 20122 Milan, Italy; 3 Department of Restorative, Preventive and Pediatric Dentistry, University of Bern, Freiburgstrasse 7, 30128 Bern, Switzerland; 4 Graduate School for Health Sciences, University of Bern, Freiburgstrasse 7, 30128 Bern, Switzerland

**Keywords:** molar-incisor hypomineralization (MIH), tooth extraction, tooth migration, space closure, pediatric dentistry

## Abstract

Background: Molar–incisor hypomineralization (MIH) represents a challenging dental condition, often requiring complex and invasive interventions. In severe cases, extraction of the first permanent molars (FPM) is frequently indicated. However, methods to assess the effectiveness of this approach in promoting spontaneous mesialization of the second permanent molars (SPM) through Orthopantomography (OPT), as well as the factors influencing this process, remain unexplored. Aim: This perspective study aimed to assess the effectiveness of novel radiograph-based methods for assessing spontaneous SPM mesialization after FPM extraction and to identify clinical and developmental factors associated with complete space closure. Methods: OPTs of 19 patients (12 males, 7 females; age range 6.2–13.8 years) who underwent extraction of 36 FPM due to severe MIH were analyzed by 2 operators. Pre- and post-extraction OPTs were evaluated using specifically developed geometric measurements of distances and angles to quantify SPM movement. Variables considered included patient age at extraction, dental developmental stage (Demirjian index), observation period, and presence or absence of the third permanent molar (TPM). The reliability of the proposed measurements evaluated through ICC (Intraclass Correlation Coefficient) resulted in values above 0.9, confirming excellent intra- and inter-operator reliability. Results: Complete or quite-complete mesialization (target scores 0–1 and ≤33% residual space) occurred in 78.6% of maxillary and 73.7% of mandibular sites. Maxillary SPMs showed more favorable spontaneous movement, exhibiting substantial uprighting (e.g., from −32° to 2°) and a higher frequency of complete or near-complete space closure (78.6% vs. 73.7% in the mandible). Developmental stages D–E of SPM were associated with successful outcomes. Conclusions: Early extraction of severely compromised first permanent molars (FPMs) can represent an effective treatment option to promote spontaneous mesialization of SPMs, particularly in the maxilla. The proposed radiograph-based measurement method demonstrated high consistency and reproducibility. Overall, this novel measurement approach may serve as a reliable and valuable tool for future clinical and research applications.

## 1. Introduction

Molar–Incisor Hypomineralization (MIH) is an increasingly recognized concern in pediatric dentistry. By definition, MIH affects the first permanent molars and permanent incisors, leading to weakened enamel and heightened sensitivity. Teeth affected by MIH are more prone to dental caries and early structural breakdown, posing significant challenges for both prevention and treatment [1,2,3]. For managing MIH an early diagnosis and an individualized approach taking into account the severity of the condition, the patient’s age, and the potential psychological impact is required [4,5]. The most commonly recommended treatments for MIH are non-invasive or minimally invasive treatments, with consideration given to the child’s age and the expected longevity of their permanent teeth. Conservative approaches can help reduce the risk of caries and protect the weakened enamel. In more severe cases, extensive restorations, protective crowns, resin infiltrations, and orthodontic bands may be necessary to provide additional support to affected molars and ensure long-term tooth stability [6,7]. Even with all the progress in treatment options, managing MIH still faces some difficulties, such as patient cooperation and treatment survival. Restorative treatments for MIH often have a high failure rate due to the structural and compositional defects of the affected enamel. The enamel in MIH teeth is softer, more porous, and less mineralized than normal, which reduces its ability to bond effectively with restorative materials and makes it more prone to fracture and wear. These challenges contribute to the frequent breakdown of restorations, highlighting the need for ongoing research and attention to this increasingly relevant condition [8,9,10].

In severe MIH cases, it might be necessary to extract the first permanent molars, especially if there is a significant structural loss, severe pain, or restoration failure. When performed at the appropriate developmental stage, typically between 8 and 10 years of age, before the eruption of the second permanent molars, this approach can promote spontaneous mesialization of the SPM into the extraction space [11]. This natural movement may reduce or even eliminate the need for complex orthodontic interventions. However, the predictability and extent of this mesialization process are influenced by several factors, including patient age at extraction, stage of dental development, and the condition of the surrounding alveolar bone.

Several studies have reported favorable outcomes following early extraction of compromised FPMs, showing that spontaneous mesial movement of the SPM is more predictable in the maxilla than in the mandible [12]. Nonetheless, issues such as molar tipping, rotation, or altered eruption paths of adjacent teeth have also been described. It has been reported that extractions carried out during Demirjian’s root development stages E and F are associated with higher success rates [12,13,14].

Ortopantomography (OPT) remains one of the most commonly used tools for evaluating dental development and post-extraction changes. It provides a comprehensive view of the dentition and surrounding structures at a relatively low radiation dose. However, OPT imaging presents several limitations related to patient positioning and projection geometry [15]. Despite these challenges, studies have shown that ratio-based assessments of vertical dimensions and tooth angulations on OPT remain consistent when repeated on the same patient at different time points [15].

Despite their widespread clinical use, there is currently only one recently proposed standardized or validated method for assessing SPM eruption pattern on OPT only in children aged 11 years [16]. Quantifying these movements accurately is essential to evaluate treatment effectiveness and to identify factors that may influence the success of space closure [17].

In this study, OPTs were used to assess the spontaneous movement of second permanent molars following the extraction of severely MIH-affected first permanent molars. Given the absence of a standardized method for evaluating mesial migration and angulation of SPMs on OPTs, two different measurement approaches are proposed and validated. Furthermore, the study explores clinical and developmental factors associated with effective space closure.

## 2. Materials and Methods

A prospective study was conducted using OPTs of pediatric patients aged 6 to 14 years that underwent the extraction of first permanent molars (FPM) severely affected by molar–incisor hypomineralization (MIH). Two different methods are presented and validated: one for assessing the spontaneous mesial migration and one for assessing inclination of second permanent molars (SPM). This study was conducted in accordance with STROBE (STrengthening the Reporting of OBservational studies in Epidemiology) guidelines and the ethical principles of the Declaration of Helsinki. Consent was obtained from parents/legal guardians for all subjects.

A total of 35 patients were initially screened; after applying the prespecified inclusion and exclusion criteria, 19 patients (12 males, 7 females) and 36 extraction sites were included in the final analysis.

Inclusion criteria:

• Growing patients aged between 6 and 14 years at the time of first permanent molar (FPM) extraction.
• Extraction of FPM due to severe molar incisor hypomineralization (MIH) with or without advanced carious lesions.

Exclusion criteria:

• Patients with systemic diseases or craniofacial anomalies that could affect dento-skeletal growth.
• Use of orthodontic appliances influencing the natural movement of teeth.
• Failure to attend follow-up visits despite repeated contact attempts.

The OPTs were collected from the databases of ASST Santi Paolo e Carlo Hospital and Fondazione IRCCS Ca Granda Ospedale Maggiore Policlinico in Milan. Digital OPT were acquired using a standardized exposure protocol on the same radiographic unit (Ortophos S 2D/3D, Dentsply Sirona, Charlotte, NC, USA). The focal distance and magnification were automatically determined by the system according to patient positioning to ensure reproducibility. Calibration and measurement accuracy were verified using the built-in digital reference scale provided by the acquisition software.

The OPTs collected and analyzed were taken before and after the extraction:

- Pre-extraction: assessment of the initial inclination and eruption stage of the SPM.
- Post-extraction follow-up: evaluation of the degree of mesialization and inclination of the SPM.

The data recorded for each patient included:

- Demographic information (sex, age);
- Medical history;
- Date of initial OPT (T0) and subsequent OPTs (T1, T2, etc.);
- Date of tooth extraction.

The geometric analysis of the OPTs was performed using an iPad Pro with Keynote software (version 14.4), integrated with a physical protractor to measure distances and angles.

Since a novel method to assess the degree of space closure following first permanent molar extraction was proposed, the sample size calculation was based on the expected reliability of the measurement. Assuming a two-rater design and targeting an intraclass correlation coefficient (ICC) of 0.80 against a null value of 0.60, with a one-sided α = 0.05 and 80% power, the required sample size was estimated at 46 teeth evaluations. Allowing for potential image exclusion or unreadable cases (10%), the planned enrolment was set at around 50 teeth evaluations to ensure adequate statistical power. To assess intra- and inter-operator reliability, the radiographic images were independently evaluated twice by the same operator, with a two-week interval between sessions, and once by two different operators. ICC (two-way random-effects, absolute-agreement) and Bland–Altman plots were used to quantify repeatability and agreement.

**Figure 1:**
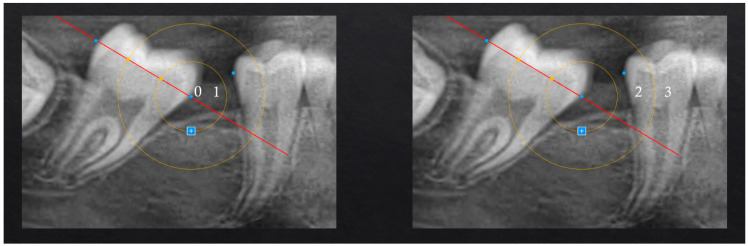
Values assigned to each target area: 0 = excellent, 1 = acceptable, 2 = not acceptable, 3 = poor.

The OPT evaluation included:

- Identification of anatomical landmarks (mesial and distal equators of the SPM and Second Permanent Premolar (SPP), and inter-radicular points of the central upper incisors).
- Construction of reference lines (lines drawn between landmarks and divided into segments to determine the molar position).
- Construction of the geometric target (as illustrated in Figure 1), using concentric circles centered on the mesial equator of the SPM to define the four-level target scale.

An inter-equatorial line was drawn connecting the mesial and distal equators for each SPM adjacent to the extraction sites.

By placing the protractor on the mesial equator of the SPM, concentric circles with increasing radii were drawn to create a target:

• First circle: radius extending to the first third of the line.
• Second circle: radius extending to two-thirds of the line.

The thickness of the circle lines was set to 0.25 pt.

The geometric target produced a four-level ordinal scale used to classify the degree of space closure:

• Score 0: complete closure;
• Score 1: near-complete closure;
• Score 2: partial closure;
• Score 3: minimal or absent closure.

These values correspond to the four concentric target areas described in Figure 1 and were used consistently for all categorical assessments.

**Figure 2:**
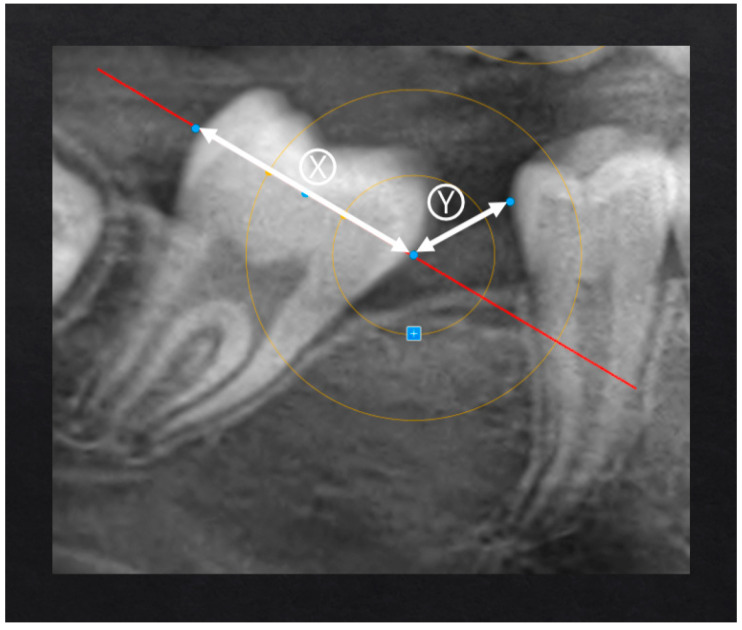
Ratio between the space between the mesial equator of the SPM and the distal equator of the SPP (Y) and the inter-equatorial length of the SPM (X).

**Figure 3:**
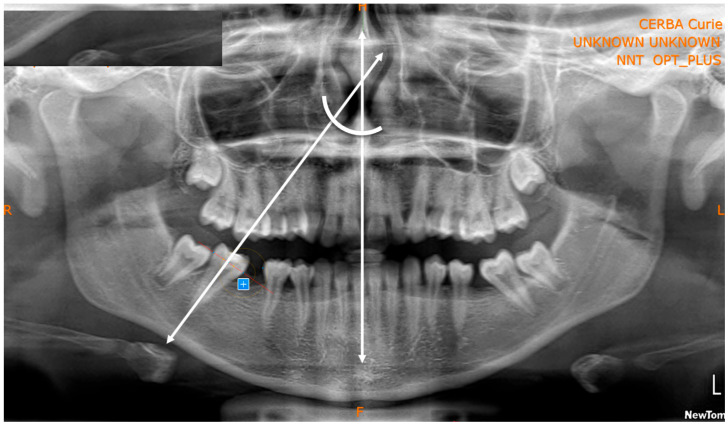
Representation of the lines used for the angular measurement of the second permanent molars.

- Evaluation of the SPM position and space closure (classification of the final position of the SPM based on the geometric target) to assess the movement from a quantitative point of view (Figure 1 and Figure 2).
- Measurement of distances and angles to assess the SPM inclination (measurements were taken using the protractor directly on the iPad screen to ensure precision and replicability) from a qualitative point of view (Figure 3).

The spontaneous movement of SPM was classified as successful when a complete or near-complete closure of the post-extraction space was observed (target scores 0–1, corresponding to ≤33% residual space). The evaluation of crown angulation was observational, as no standardized reference parameter is available in the literature.

Cases with target scores ≥ 2 or excessive mesial tipping were classified as unsuccessful.

**Figure 4:**
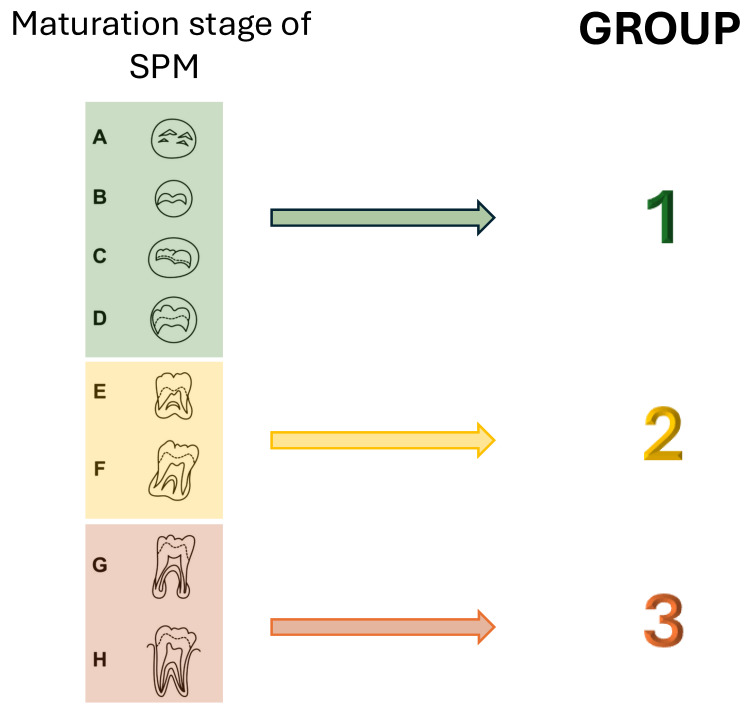
Representation of patient distributions in 3 groups based on the maturation stage of the second permanent molar according to Demirjian.

The following variables have been analyzed:

- Target area (Figure 1);
- Inter-equatorial distance of the SPM;
- Interdental distance SPM-SPP;
- Ratio of interdental distance SPM-SPP to the inter-equatorial distance of the SPM (Figure 2);
- Space closure percentage;
- Residual space percentage;
- Inclination of the SPM (Figure 3) based on validated inclination measurements [18,19];
- Observation times;
- Eruption phase of the SPM (Figure 4).

All variables, except for the eruption phase of the SPM, were repeated for each subsequent follow-up OPT.

Figure 1 and Figure 2 show the target and ratio, parameters used to validate the reliability of the proposed measurement method. The target and ratio both describe the post-extraction space closure from complementary perspectives: the ratio expresses the amount of residual space as a proportion of the original extraction gap, whereas the target converts this information into discrete scores that facilitate clinical interpretation of the outcomes.

The following criteria were used to verify the correspondence between the two methods:

• Target score 0: The ratio must be equal to or very close to 0.
• Target score 1: The ratio value must not exceed 33.33%.
• Target score 2: The ratio value must not exceed 66.66%.
• Target score 3: The ratio value is greater than or equal to 66.67%.

The calculated ratio values consistently matched the estimated target scores, thus validating the mutual reliability of the two measurement methods.

For time-based evaluation, each extracted site (n = 36) was assigned to one of three follow-up groups:

• T1: <18 months;
• T2: 19–35 months;
• T3: ≥36 months.

This grouping was used for all time-trend analyses.

**Statistical Analysis**

The molars studied were divided into 3-time groups:

- T1: Follow-up OPT < 18 months;
- T2: Follow-up OPT from 18 to 35 months;
- T3: Follow-up OPT > 36 months.

Additionally, the molars were divided based on the Demirjian development stages [20], as follows:

- Group 1: Stages A, B, C, D;
- Group 2: Stages E, F;
- Group 3: Stages G, H.

For the analysis of inclinations and movements over time, random intercept linear regression models were used to account for within-subject variables. Stata software, version 18, was used.

## 3. Results

A total of 19 patients (12 males, 7 females) with 36 extracted first permanent molars and 55 target evaluation were included in the analysis. All outcomes were therefore evaluated per extraction site (n = 36)

A total of 36 FPMs, who had been advised to undergo extraction of at least one non-restorable first molar, were assessed according to the inclusion and exclusion criteria. Nineteen patients (12 males and 7 females) met the eligibility criteria and were included in the study. Twelve patients were excluded for the following reasons:

• 11 lacked final radiographic documentation.
• 1 had undergone fixed orthodontic treatment for aesthetic reasons.
• 1 did not consent to extraction.
• 3 became unreachable.

The patients involved in the study presented a total of 36 MIH-affected first molars that were extracted, distributed as follows:

• 8 upper right FPMs;
• 9 upper left FPMs;
• 9 lower left FPMs;
• 10 lower right FPMs.

The number of extracted molars per patient was as follows:

• 10 patients with 1 extracted molar;
• 5 patients with 2 extracted molars;
• 4 patients with 4 extracted molars.

One molar (tooth 47) was excluded due to the placement of a space maintainer. However, the patient was not excluded from the study, as the tooth 27 could still undergo mesialization.

The patients’ age at the time of extraction ranged from 6.2 to 13.8 years.

A total of 55 targets were analyzed, 55 residual space ratios and inter-equatorial distances were calculated, and 91 SPM angular measurement of inclination were recorded.

The reliability of the measurements was assessed using the Intraclass Correlation Coefficient (ICC) with corresponding 95% confidence intervals (95% CI). All parameters demonstrated excellent reproducibility, both within and between operators (Table 1). All ICC values in this study were above 0.9, confirming excellent intra- and inter-operator reliability. The measurements are highly consistent and reproducible, both when repeated by the same operator and when performed by different operators.

**Figure 5:**
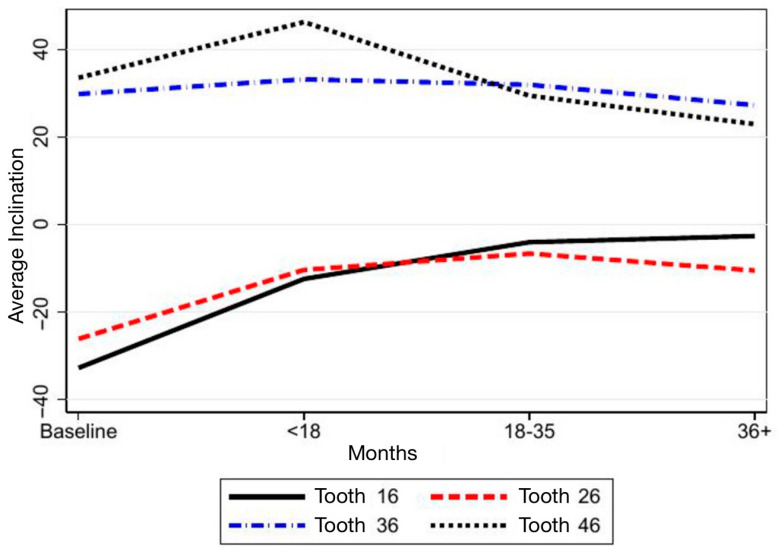
Representation of the inclination trend of each second permanent molar (SPM) over time.

**Figure 6:**
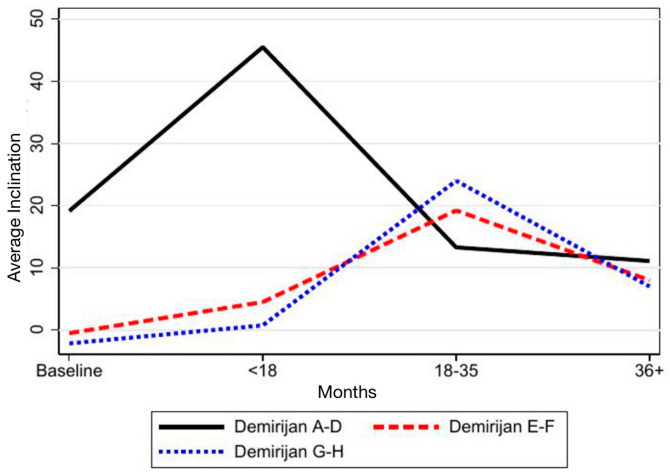
Time-based representation of the inclination changes of second permanent molars (SPMs) within each Demirjian group.

Regarding SPM inclination, in the maxillary arch, tooth 17 showed a marked change in inclination over time, shifting from −32° to 2°, similar to the trajectory observed for tooth 27. Mandibular teeth 37 and 47 did not exhibit comparable changes (Table 2; Figure 5). When analyzed by developmental groups, a significant evolution in inclination emerged in Groups 2 and 3 (coefficients 8 and 7, respectively), whereas Group 1 (Demirjian stages A–D) showed no meaningful change (Table 3; Figure 6).

**Figure 7:**
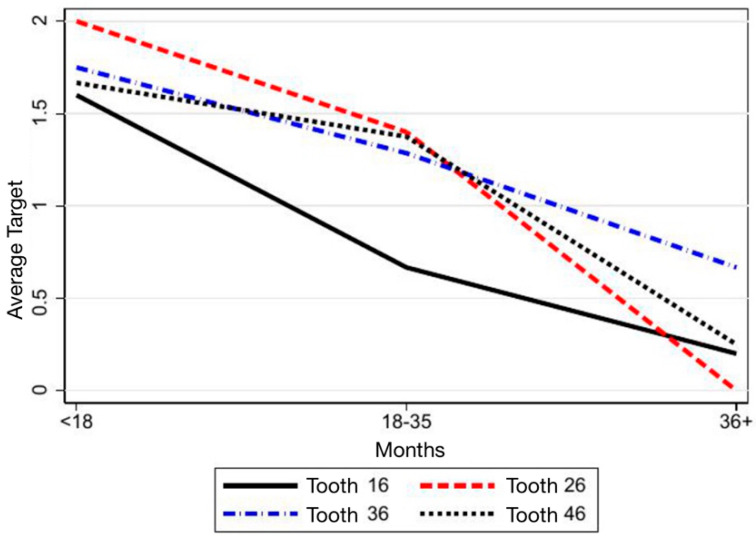
Representation of the target score trend for each second permanent molar (SPM) over time.

**Figure 8:**
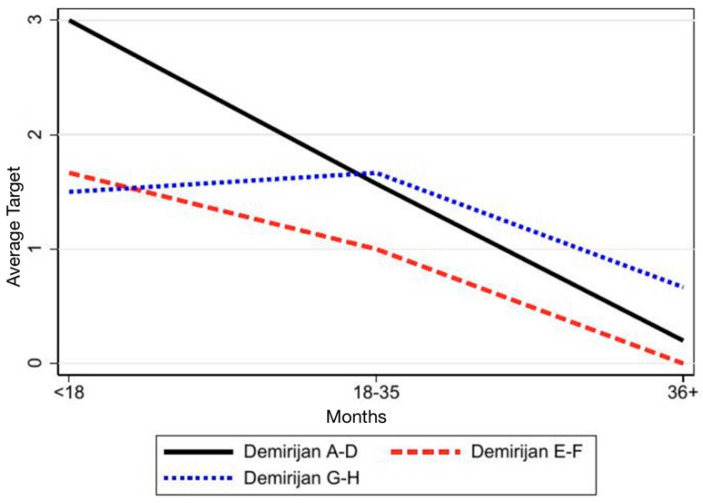
The graphic shows the teeth categorized into the three Demirjian groups and illustrates how their position relative to the target changes over time.

Consistently, assessment of tooth movements trough target values showed a decline across time for all teeth examined except for 36, which displayed an uncertain trend (Table 4; Figure 7). In Groups 2 and 3, target values decreased, while Group 1 again showed no significant evolution (Table 5; Figure 8).

**Figure 9:**
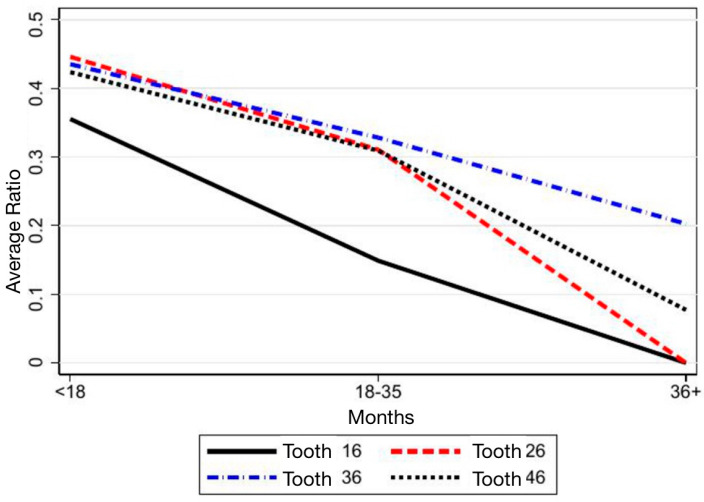
Trend over time of the ratio between the inter-equatorial distance of the SPM and the distance from the mesial equator of the SPM to that of the SPP.

**Figure 10:**
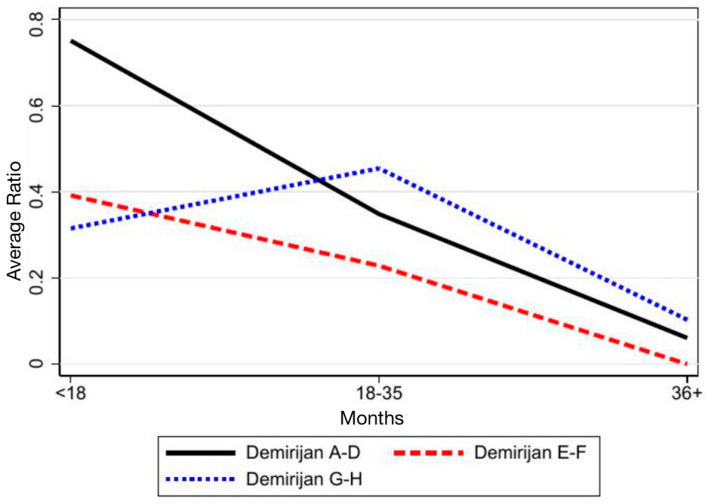
Variation of the ratio over time in teeth categorized into the three Demirjian groups.

Figure 9 and Figure 10 show a progressive decrease in the ratio over time for most SPMs (more pronounced in the maxilla) which is consistent with progressive space closure. The decrease is more regular and clinically meaningful in Demirjian groups 2–3, whereas group 1 shows smaller changes due to lower maturation. Overall, these patterns support the robustness of the proportional method and align with the target-based results.

Analyzing spontaneous mesialization of second permanent molars (SPMs), the upper arch achieved a 78.57% success rate (14/17 molars). The failures were confined to two maxillary molars, both extracted at Demirjian stage E and followed for only 8 months. In the lower arch, the success rate was 73.68% (14/19 molars). Failures occurred in five patients, two of which had both lower first permanent molars extracted in the absence of third-molar (TPM) buds and were followed for 23 months. Additional failures involved 3 subjects at various Demirjian stages with follow-ups from 19 to 34 months.

## 4. Discussion

The present study investigated the spontaneous mesial migration of second permanent molars following the extraction of first permanent molars severely affected by molar-incisor hypomineralization. A novel radiographic approach, based on geometric targets and proportional ratios on OPT was introduced to evaluate these movements. Although OPT provide two-dimensional images with a degree of distortion, they remain the most practical and ethically acceptable imaging method in pediatric dentistry.

By employing ratios instead of absolute distances, the influence of magnification and positional variability inherent to different panoramic units was reduced, enabling more standardized comparisons across radiographs. In addition, the complementary geometric target allowed a categorical assessment of space closure consistent with the ratio values, improving reproducibility and facilitating clinical interpretation.

Crown-based reference axes were selected to minimize the variability associated with root morphology, which is particularly relevant in developing teeth affected by MIH. While this choice may not perfectly capture radicular inclination, it reliably reflects the clinically meaningful changes and remains applicable even when apices are incomplete or poorly defined. This method represents a reasonable balance between precision and radiation safety, making it suitable for longitudinal studies in pediatric populations where minimizing exposure is essential.

The findings of this study showed a more favorable inclination and a higher frequency of spontaneous space closure in the maxillary arch compared with the mandible. This difference can be explained by the physiological eruption pattern of the second molars. In the maxilla, these teeth usually begin eruption with a distal inclination due to posterior crowding, and their subsequent mesial movement represents a normal phase once space becomes available after FPM extraction. In contrast, mandibular molars generally erupt along a more vertical path and tend to develop mesial tipping later as part of their natural forward drift. Moreover, the denser cortical bone of the mandible may further limit spontaneous uprighting compared with the maxilla [14,21].

Several authors have explored additional variables that may influence spontaneous space closure. Patel et al. proposed a radiographic “toolkit” for evaluating mandibular SPM angulation and third molar presence, suggesting that mesial angulation and third molar presence improved the likelihood of closure in the mandible [22]. However, Nordeen et al. and Ciftci et al., failed to validate this model, indicating that developmental stage and age are more reliable predictors than angulation alone [23,24]. The present study supports this interpretation: according to reported results spontaneous uprighting and mesialization occur predictably when extractions are performed early, while excessive mesial tipping or incomplete uprighting tend to appear in later stages.

In terms of success, the findings are consistent with previous reported by Nordeen et al., indicating an 82% success rate of spontaneous closure in the maxillary arch compared with 51% in the mandibular arch, with developmental stage and chronological age appearing to play a greater role than initial molar angulation or the presence of third molars [23]. Similarly, Teo et al. and Brusevold et al. described complete spontaneous eruption of the maxillary second molars into the extraction space, while nearly 30% of mandibular cases required orthodontic space closure [12,25]. These results further confirm that spontaneous mesialization is highly predictable in the maxilla but less consistent in the mandible, likely due to differences in bone density and eruptive trajectory. The present results therefore reinforce the concept that extractions before the onset of second molar root formation enhance the chance of spontaneous space closure and favorable molar angulation. Moreover, Hamza et al., in a recent systematic review and meta-analysis, confirmed that the success rate is better in the maxilla compared with the mandible, and emphasized that extractions before the completion of SPM root formation substantially increase the probability of spontaneous substitution [26]. Current findings, when considered alongside previous research, reinforce that early extraction of severely affected FPMs (ideally at Demirjian stages D or E) can favor predictable spontaneous mesialization, particularly in the maxilla [22,24].

From a clinical standpoint, the ratio-target model proposed in this study provides a reliable, low-dose approach for assessing both the extent of space closure and the quality of tooth angulation during spontaneous movement. This standardized method may facilitate the identification of the optimal timing for first molar extraction in children affected by MIH and aid in anticipating potential orthodontic requirements. Further investigations incorporating three-dimensional imaging and larger sample sizes are warranted to validate crown-based angular measurements relative to root inclinations and to refine predictive parameters for clinical application.

The proposed methodological contribution adds a standardized, low-dose tool for assessing post-extraction space closure in children aged between 6 to 14 years, potentially improving treatment planning and interdisciplinary communication between pediatric dentists and orthodontists.

Limitations 

This study has several limitations that should be acknowledged. Variability in the timing of radiographic examinations among participants may have affected the extent of the observed second permanent molar movement. Moreover, potential differences related to sex and age could not be fully accounted for due to the limited sample size. The relatively small cohort of 19 subjects also constrains the generalizability of the results. Further investigations involving larger and more homogeneous populations are required to validate and expand upon these preliminary findings.

## 5. Conclusions

Spontaneous mesialization of SPMs occurs after extraction of compromised FPMs in children with severe MIH, with a more favorable movement in the maxilla than in the mandible. Earlier extractions, before the eruption of SPMs, are usually associated with more predictable space closure and more controlled mesial angulation.

The upper molars appear to have a better quality of movement compared to the lower ones, likely because they start from a more favorable position. The angular movement of mesial tipping acts as a positive compensatory response for the upper molars, which tend to straighten up, whereas it seems to negatively affect the lower molars, causing them to tilt forward.

The influence of craniofacial growth patterns and occlusal relationships should also be considered, as skeletal discrepancies may affect molar movement and space closure dynamics.

Future studies with larger samples to explore whether there’s a true association between cephalometric data and the degree of inclination of the molar crowns adjacent to extraction sites, particularly in cases involving teeth affected by caries or enamel defects. Research should also address strategies to not only re-establish proper contact points between teeth adjacent to the extraction area but also to ensure the correct alignment of both crowns and roots.

Moreover, insights from studies on space management after premature molar loss highlight the importance of early interceptive approaches to guide spontaneous or assisted space closure effectively [27].

Ultimately, the early extraction of compromised FPMs in case of severe MIH may turn a liability into a biologically assisted opportunity for space closure, especially in the maxilla. From a clinical standpoint, timely diagnosis combined with interdisciplinary treatment planning can facilitate natural space closure and minimize the need for complex orthodontic interventions.

## Figures and Tables

**Table 1:** ICC results on measures reliability.

ICC				
	Intra-Operator		Inter-Operator	
	ICC	95CI	ICC	95CI
**INTER-EQUATORIAL DISTANCE OF THE SPM**	0.98	0.96; 0.99	0.97	0.94; 0.99
**INTERDENTAL DISTANCE—SPP**	0.99	0.99; 1.00	0.98	0.97; 1.00
**RATIO**	0.99	0.99; 1.00	0.99	0.98; 1.00
**SPM INCLINATION**	0.97	0.96; 0.99	0.95	0.92; 0.99

**Table 2:** Mean values (in sexagesimal degrees) of the average tipping of each quadrant recorded in the initial OPT (T0) and in subsequent OPTs taken at <18 months, 18–35 months, and >36 months.

	T					
EXTRACTED SPM	T0	T1	T2	T3	Coefficient	_95%_CI
**16**						
**N**	8	5	3	5		
**INCLINATION**	−32.75	−12.40	−4.00	−2.60	11.40	[7.87; 14.93]
**26**						
**N**	9	6	5	2		
**INCLINATION**	−26.11	−10.33	−6.60	−10.50	12.11	[8.73; 15.49]
**36**						
**N**	9	4	7	3		
**INCLINATION**	29.89	33.25	32.00	27.33	−0.58	[−4.66; 3.49]
**46**						
**N**	10	3	8	4		
**INCLINATION**	33.60	46.33	29.50	23.00	−3.43	[−6.61; 0.24]

**Table 3:** Mean values (in sexagesimal degrees) of the inclinations measured in each Demirjian group, recorded in the initial panoramic radiograph (T0) and in subsequent radiographs taken at <18 months, 18–35 months, and >36 months.

	T					
D EMIRJIAN	T0	T1	T2	T3	Coefficient	95%CI
**GROUP 1**						
**N**	7	2	7	10		
**TARGET**	19.14	45.50	13.29	11.10	−1.78	[−10.55; 6.99]
**GROUP 2**						
**N**	22	12	13	1		
**TARGET**	−0.50	4.50	19.23	8.00	7.88	[−0.69; 16.46]
**GROUP 3**						
**N**	7	4	3	3		
**TARGET**	−2.14	0.75	24.00	7.00	7.00	[3.26; 10.75]

**Table 4:** Mean target outcome values for each quadrant measured on follow-up panoramic radiographs (OPTs) taken at <18 months, 18–35 months, and >36 months.

	T				
DE MIRJIAN	T1	T2	T3	Coefficient	95%CI
**16**					
**N**	5	3	5		
**INCLINATION**	1.60	0.67	0.20	−0.68	[−0.95; −0.38]
**26**					
**N**	6	5	2		
**INCLINATION**	2.00	1.40	0.00	−0.91	[−1.49; −0.33]
**36**					
**N**	4	7	3		
**INCLINATION**	1.75	1.29	0.67	−0.54	[−1.19; 0.12]
**46**					
**N**	3	8	4		
**INCLINATION**	1.67	1.38	0.25	−0.74	[−1.40; −0.76]

**Table 5:** Mean target outcome values for each Demirjian group measured on follow-up OPTs taken at <18 months, 18–35 months, and >36 months.

	T				
D EMIRJIAN	T1	T2	T3	Coefficient	95%CI
**GROUP 1**					
**N**	2	7	10		
**TARGET**	3.00	1.57	0.20	−1.58	[−2.09;−1.06]
**GROUP 2**					
**N**	12	13	1		
**TARGET**	1.67	1.00	0.00	−1.02	[−1.68;−0.36]
**GROUP 3**					
**N**	4	3	3		
**TARGET**	1.50	1.67	0.67	−0.41	[−0.77;−0.43]

## Data Availability

The data presented in this study are available from the corresponding author upon reasonable request. During the preparation of this manuscript/study, the authors used AI to check the text and table for errors. The authors reviewed and edited the results and take full responsibility for the content of this publication. request, after the signature of a formal data sharing agreement in anonymous form, from the corresponding author because they are protected by privacy.

## Abbreviations

The following abbreviations are used in this manuscript:

MIH	Molar-Incisor Hypomineralization.
FPM	First Permanent Molar
SPM	Second Permanent Molar
OPT	Oprtopantomography
CBCT	Cone-Beam Computed Tomography.
SPP	Second Premolar
ID	Identifier
